# MANTA closure device shows promise in transfemoral transcatheter aortic valve replacement

**DOI:** 10.1007/s12471-020-01511-0

**Published:** 2020-10-28

**Authors:** J. Vendrik, J. Baan Jr.

**Affiliations:** Department of Cardiology, Heart Centre, Amsterdam UMC, University of Amsterdam, Amsterdam Cardiovascular Sciences, Amsterdam, The Netherlands

In this issue of the *Netherlands Heart Journal*, Halim et al. report the results of a single-centre study assessing the efficacy and safety of the use of the MANTA large-bore vascular closure device in transfemoral transcatheter aortic valve replacement (TF-TAVR) [1]. The MANTA closure device showed promise in these patients, although a considerable proportion of them had access-site-related vascular complications. The cohort was small, but the authors used a well-designed study protocol and performed an adequate analysis.

The outcomes of earlier studies [2–7] are in line with those reported by Halim et al., describing a prevalence of access-site-related vascular complications—as defined by the Valve Academic Research Consortium-II criteria [8]—ranging from 2–14%. The authors correctly identified a steep learning curve when using the MANTA device, even though they did not analyse this [1]. Hoffman et al. reported a vast decline of complications after the first 25 cases [4]. This is especially useful information for TAVR centre start-ups in an era in which TAVR is expanding to a broader (i.e. low-risk) population, as the learning curve for suture-based vascular closure devices (i.e. ProGlide and Prostar) is thought to be more extensive in general. The different outcomes for the newer MANTA closure device and the traditionally used suture-based vascular closure devices may very well be explained by each device’s advantages, disadvantages and proper indications, which influence patient selection and therefore outcomes.

Earlier reports have focussed on the occurrence and eventually prevention of access-site-related vascular complications. First, Van Kesteren et al. identified the ‘sheath-to-iliofemoral artery ratio’ to be the only strong predictor of vascular complications, while adding that all vascular complications influence survival [9]. Hence, even minor complications, such as access site bleeding, should be avoided when possible.

Second, focussing on the MANTA closure device, Moccetti et al. recently hypothesised three distinct mechanisms of its failure: (a) elevation of the toggle may lead to occlusion of the artery in vessels with narrow femoral artery diameters; (b) incomplete apposition of the plug may lead to perivascular, potentially retroperitoneal bleeding; or (c) the formation of a pseudoaneurysm may occur [3]. This knowledge could further lower the prevalence of access-related complications surrounding TF-TAVR, in addition to gaining extensive experience in using these kinds of closure devices.

The aforementioned knowledge is much awaited for two reasons: (i) vascular complications are still the most common complications after TF-TAVR; and (ii) adequate vascular closure without complications could ensure both early ambulation and early discharge. Early ambulation can prevent other avoidable complications, such as postoperative delirium and postoperative infections [10], favour patient satisfaction and ultimately enable early discharge. When properly employed, an early discharge strategy can improve the cost effectiveness of TAVR and ultimately improve patient satisfaction, without imposing additional risks for the patient [11–14].

The aforementioned parameters, i.e. early ambulation and early discharge, greatly influence cost effectiveness. At this moment, the MANTA device is probably more expensive than the commonly used ProGlide suture-based vascular closure device. To assess the true cost effectiveness and comparability of large-bore closure devices and conventional suture-based systems, outcome measures of future studies should not only include the number of successful closures, access-site-related vascular complications and bleedings, but also the effect of these numbers on subsequent hospitalisation.
